# Modelling of the Process of Extrusion of Dry Ice through a Single-Hole Die Using the Smoothed Particle Hydrodynamics (SPH) Method

**DOI:** 10.3390/ma15228242

**Published:** 2022-11-20

**Authors:** Krzysztof Wałęsa, Jan Górecki, Maciej Berdychowski, Aleksandra Biszczanik, Dominik Wojtkowiak

**Affiliations:** Institute of Machine Design, Poznan University of Technology, 61-138 Poznań, Poland

**Keywords:** extrusion, compression, SPH, die, dry ice, carbon dioxide, CO_2_

## Abstract

This article presents the outcome of research on modelling the process of the extrusion of crystalline dry ice. The purpose of this process is to densify the material and obtain pellets of several millimeters in diameter. This reduces the sublimation rate in ambient conditions of the material whose temperature in a solid state is 195 K. A lower sublimation rate means a reduction of the loss of product in its final applications, which include refrigeration and reduction of atmospheric emissions of gaseous CO_2_. A ram-type extruder was considered in this analysis, in which dry ice was extruded through a single-hole die of varying geometry. The article presents the results of numerical analyses of the extrusion process, using a simulation method based on the Smoothed Particle Hydrodynamics (SPH) approach. The results from simulations were verified by the experimental data in terms of the maximum force required to complete the process, in order to assess the applicability of the proposed method in further research on dry ice compression.

## 1. Introduction

The issue of reuse of waste generated throughout the entire lifecycle of products is becoming a more important issue in the area of process engineering [1,2,3,4]. It is often practicable to recover waste materials generated during various production processes. Górecki observed that huge amounts of carbon dioxide are emitted into the atmosphere during production of ammonia compound, due to the high degree of purity of waste material from which it is recovered [5]. This material is condensed through compression and then stored at a pressure of 20 bar and a temperature of 216 K to 250 K [6,7]. Rapid expansion of atmospheric pressure results in a liquid-solid phase transition. In ambient conditions, crystallised carbon dioxide (CCD) has a temperature of 195 K and changes its state in the process of sublimation [8,9].

These peculiar properties are desired in a number of production processes, including refrigeration [10,11] and cleaning of surfaces [12,13]. These processes utilise the two specific characteristics of CCD, i.e., low temperature and sublimation. CCD obtained through expansion is a very fine grain material of ca. 100 μm size of particles [14,15]. The high specific surface characteristics of dry ice snow results in a high sublimation rate, affecting the process efficiency in refrigeration application [14]. For this reason, the most common commercial form of dry ice are pellets of 3–16 mm in diameter featuring a lower sublimation rate [16].

**Figure 1:**
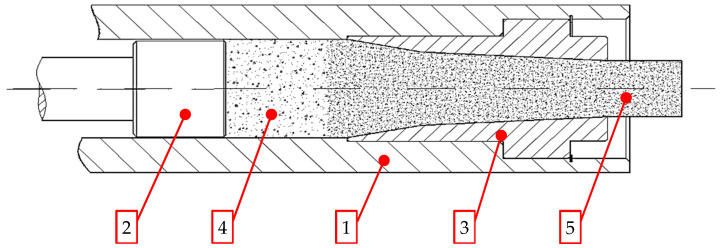
Main parts of the ram-type extruder: 1—compression cavity, 2—ram, 3—die, 4—loose dry ice, 5—compressed dry ice [17].

Dry ice snow is compressed in ram-based extrusion machines. The working system of such machines is shown schematically in Figure 1. The raw material is expanded inside the compression cavity (1). Then, crystalline carbon dioxide (3) is compressed by a ram pressing it against the residual, already compressed CCD (4) that fills up the die cavity (5).

**Figure 2:**
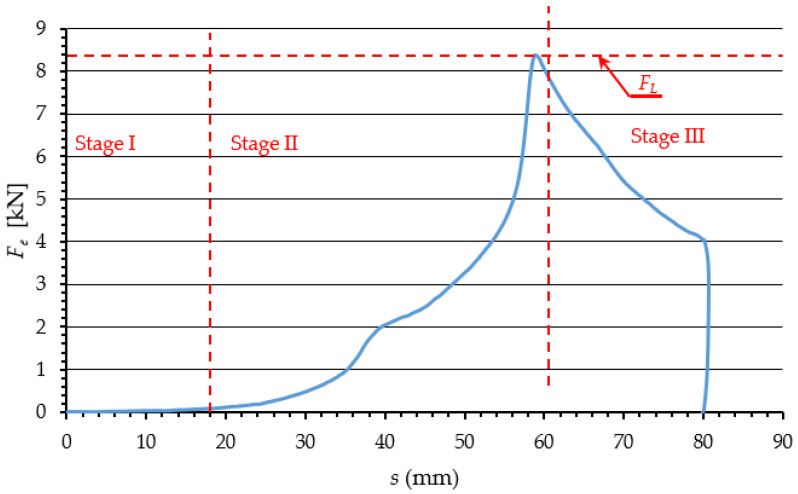
Phases of the compaction and extrusion process during one work cycle of the work system built for the purpose of this research: Phase 1—Initial compression, Phase 2—Final compression, Phase 3—Extrusion [21].

The graph in Figure 2 represents the change of *F*_e_ as a function of ram displacement *s*, subdivided into three main phases of the change of force applied to extrude dry ice pellets.

In Phase 1, dry ice snow is compressed without any significant increase in the value of force *F*_e_. This is because the displacement of ram reduces spaces between the particles. The exerted pressure does not induce elastic or plastic strains, due to ignorable contact between the particles. In Phase 2, the particles are packed closer together and the internal friction and elastic and plastic strain come into play as a result. The touching and relative displacement of particles increases the value of the exerted force *F*_e_, due to elastic and plastic strain, and internal friction. In addition, the compressed material interacts with the compression cavity walls, due to elastic strain. This results in friction between the compressed material and the compression cavity walls, increasing the compression resistance. The dissipation of energy during compression, in cylindrical chambers with tapered ends, has been extensively described in the literature [18,19].

The value of force *F*_e_ increases until it equals the maximum resistance force of the process of compression *F*_L_. It has been demonstrated that the value of this force depends on the die parameters and the coefficient of friction between the extruded material and the side walls of the die [18]. The value of *F*_e_ decreases as the process of extrusion proceeds and the density of the material *ρ* no longer increases as a result [20].

Compression of the material reduces the distances between the particles of the material observed as an increase in density *ρ*. As the value of *ρ* increases, so do the values of Young’s modulus *E*, Poisson’s ratio *ν*, and coefficient of friction *µ* [17,20,22]. The behaviour of the compressed material in Phase 2 may be accurately simulated with Drucker-Prager/Cap, Cam-Clay, and Mohr-Coulomb models [23,24,25].

The decrease of *F*_e_ observed in Phase 3 marks the transition from compression to extrusion. The correlation coefficient defining the similarity of the *F*_e_ vs. *s* curve, and a straight-line relationship is 0.9, which indicates a proportional decrease of force in relation to displacement. This is due to the linear change in the surface area of the side walls of the compression cavity (1 on Figure 1) that comes into contact with the material. This allows us to assume the constant values of *E*, *ν*, and *μ*, and use an elastic-plastic model to represent the process in numerical studies, as it has been done in previous studies reported in the literature [26,27,28].

According to Jankowiak [29] the Smoothed Particle Hydrodynamics (SPH) method can be used to accurately describe the geometry of materials featuring high plastic strain during compression. Originally, SPH was developed to simulate the flow of fluids. Since then, it was adapted to simulate processes that involve high strains, such as compression and extrusion of solids [16,29]. Sakai observed that the accuracy of predictions with the SPH method decreases in areas close to the boundary conditions, or free surfaces [30]. Even though the predictions obtained with SPH are close to the results obtained with the conventional mesh-based method, these are subject to the assumption of appropriate spacing between particles [16].

The SPH method is a meshless way of carrying out numerical simulations, in particular, fluid dynamics, based on Lagrangian description of the modeled liquid domain. Therefore, the SPH model is characterized by the fact that individual particles, which meet the roles corresponding to the nodes of Euler’s FEM methods, move freely in terms of bonds imposed during the simulation. In this case, however, the mesh is not a restriction for their movement [28,30]. Single particles during simulated flow carry the necessary information, such as the value of speed or density. During the simulated flow, they interact with each other in accordance with the principles of fluid dynamics [31].

This calculation method is based on the theory of interpolation. Continuous distribution of such parameters as speed or liquid pressure are replaced by appropriate estimates with a certain interpolation kernel. Calculations are performed using a discreet set of a specific number of fluid particles. In the general approach, the estimation of any *F*i in a certain position of the *i* particle is described as:

(1) $$F_{i}=\sum_{j=1}^{N}m_{j}\cdot \frac{F_{j}}{\rho_{j}}\cdot W_{ij}\left(r_{ij},h_{ij}\right),$$

where *m_j_*—the mass of the *j* particle, *ρ_j_*—density of the *j* particle, and *W_ij_*—kernel function, depend on the smoothing length *h_ij_* and distance between particles *r_ij_*.

This method, using the general Equation (1) can be used to calculate typical tensor quantities, such as speed, pressure or stress. It should be noted, however, that the basis for calculations is information about density at specific points, represented by particles located in specific positions. Information about this parameter is used to estimate the physical quantity in request. Therefore, the authors decided that this method can be successfully used to estimate the force of extrusion of dry ice by the matrix, as shown by analyses of large deformations of material with elastic-plastic features.

This article presents the outputs of computer simulations of the process of dry ice extrusion using four different extrusion dies. The objective was to find the maximum value of *F*_e_ for different shapes of the extrusion die. The predicted values were compared with the experimental data to assess the accuracy of representation of the process by numerical methods, primarily SPH based. The primary goal was to develop a relatively simple technique for estimating the maximum resistance during compression of solid dry ice that could be used to maximise the process efficiency when designing new dies. To this end, it was required to find a numerical solution, for example with the application of SPH, to model the process of extrusion of dry ice without needing to use sophisticated material models, which require extensive testing to determine the material properties. With this approach it will be possible to expand the research also on other materials.

In addition, the outcome of this research allowed the assessment of the influence of die shape parameters on the maximum force applied in the dry ice extrusion process.

## 2. Materials and Methods

### 2.1. Solid Carbon Dioxide

The compressed material dry ice snow was obtained by expansion of liquid carbon dioxide stored in a sealed container at a pressure of 20 bar and at −18 °C. Liquid to solid state transition occurs in an adiabatic process when liquid carbon dioxide undergoes a rapid expansion to atmospheric pressure. This results in crystallisation. The resulting dry ice snow has a density of 550 kg/m^3^ [30].

Before the empirical tests were carried out, as part of this research, dry ice was stored in a foamed polystyrene container. The insulated container allowed the minimisation of the sublimation rate. These storage containers were also used to cool down the test assembly to a temperature close to the temperature of the tested material.

The following parameters of dry ice snow were adopted based on experiments carried out by the authors of this article, reported in previous studies [20,32]:

− Young’s modulus *E* = 881 MPa;
− Poisson’s ratio *υ* = 0.46;
− Density *ρ* = 1625 kg/m^3^;
− Input yield stress *σ_pl_* = 3.5 MPa.

### 2.2. Single-Hole Dies

**Figure 3:**
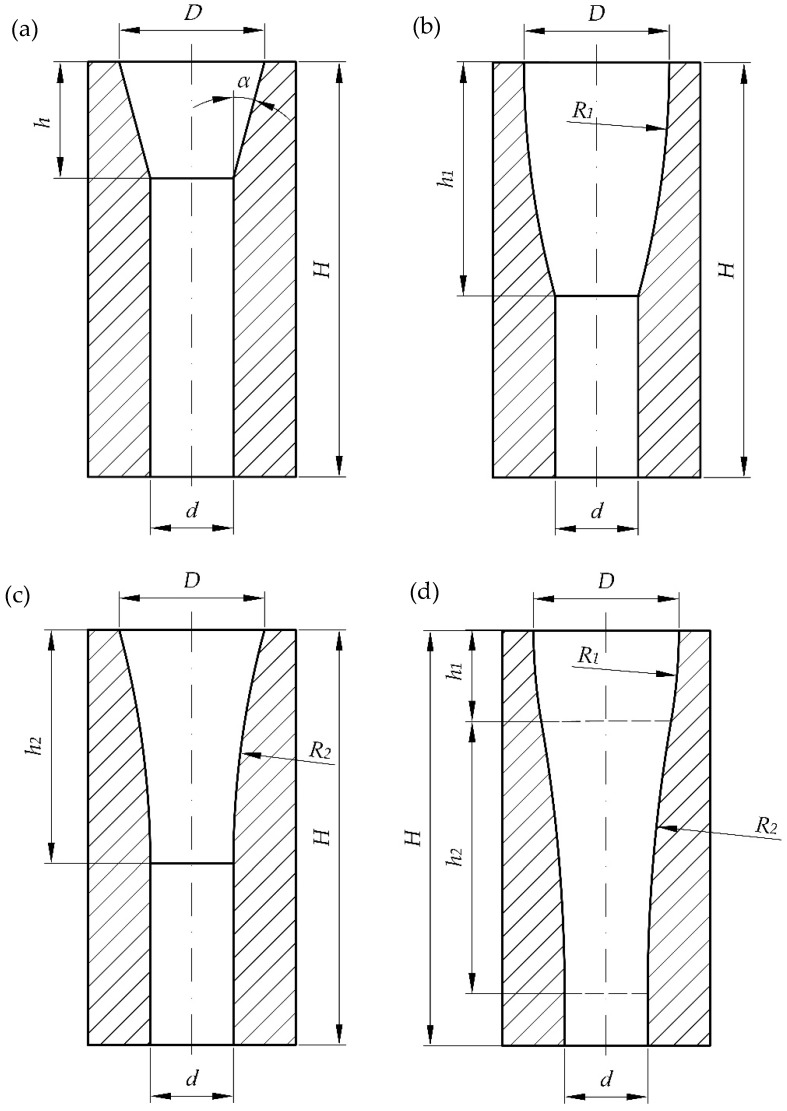
Shapes of the dies under analysis: (**a**) conical-cylindrical (CS), (**b**) with a convex spherical compression section (WK), (**c**) with a concave spherical compression section (WP), (**d**) with a convex section followed by concave section (WKWP).

The tests and numerical simulations were carried out for four different single-hole dies shown in Figure 3, each having a different inlet section. The shape of the compression cavity of the tested dies was described with geometric parameters defining its lengthwise section. The following dimensions were the same for all the dies in consideration:

− Inlet diameter *D* = 28 mm;
− Outlet diameter *d* = 16 mm;
− Overall height *H* = 80 mm.

Four shapes of the compression cavity were distinguished:

− Conical-cylindrical (CS) with a truncated cone inlet section whose shape is defined by the wall taper angle *α* and conical section height *h* (Figure 3a);
− Spherical-cylindrical (WK) with a convex frustum of a sphere defined by the sphere radius *R_1_* and height of the spherical part *h*_1_ (Figure 3b);
− Spherical-cylindrical (WP) with a concave frustum of a sphere defined by the sphere radius *R_2_* and height of the spherical part *h*_2_ (Figure 3c);
− Spherical-cylindrical (WKWP) with the inlet section made up of two spherical frustums arranged in a series, convex followed by concave. This shape is defined by the sphere radii *R*_1_ and *R*_2_ and the heights of the spherical frustums *h*_1_ and *h*_2_ (Figure 3d).

The geometric parameters of the above-described types of compression cavities (Figure 3) can be interrelated by mathematical relationships. These relationships can be written as follows:

− for CS cavity:

(2) $$\alpha =\tan^{-1}\left(\frac{\frac{1}{2}\cdot \left(D-d\right)}{h_{1}}\right),$$

− for cavities WK and WP:

(3) $$R_{1}=\frac{\frac{1}{2}\cdot \sqrt{{\left(\frac{1}{2}\cdot D-\frac{1}{2}\cdot d\right)}^{2}+h_{1}{}^{2}}}{\sin\left(\frac{180{}^{\circ}-2\cdot \tan^{-1}\left(\frac{h_{1}}{\frac{1}{2}\cdot \left(D-d\right)}\right)}{2}\right)},$$

− and for cavity WKWP, with an additional parameter *H*′ calculated as the sum of *h*_1_ and *h*_2_ as follows:

(4) $$H^{\prime }=h_{1}+h_{2},$$

we can introduce a proportionality coefficient *a*:

(5) $$a=\frac{h_{1}}{H^{\prime }},$$

and using this coefficient to relate radii *R*_1_ and *R*_2_ to the other geometric parameters of the cavity:

(6) $$R_{1}=\frac{\frac{1}{2}\cdot \sqrt{{\left(\frac{1}{2}\cdot D-\left(\frac{1}{2}\cdot D-\left(\frac{1}{2}\cdot a\cdot \left(D-d\right)\right)\right)\right)}^{2}+{\left(a\cdot H^{\prime }\right)}^{2}}}{\sin\left(\frac{180{}^{\circ}-2\cdot {\mathrm{tg}}^{-1}\left(\frac{a\cdot H^{\prime }}{\frac{1}{2}\cdot D-\left(\frac{1}{2}\cdot D-\left(\frac{1}{2}\cdot a\cdot \left(D-d\right)\right)\right)}\right)}{2}\right)}.$$

(7) $$R_{2}=\frac{\frac{1}{2}\cdot \sqrt{{\left(\left(\frac{1}{2}\cdot d+\left(\frac{1}{2}\cdot \left(1-a\right)\cdot \left(D-d\right)\right)\right)-\frac{1}{2}\cdot d\right)}^{2}+{\left(\left(1-a\right)\cdot H^{\prime }\right)}^{2}}}{\sin\left(\frac{180{}^{\circ}-2\cdot {\mathrm{tg}}^{-1}\left(\frac{\left(1-a\right)\cdot H^{\prime }}{\left(\frac{1}{2}\cdot d+\left(\frac{1}{2}\cdot \left(1-a\right)\cdot \left(D-d\right)\right)\right)-\frac{1}{2}\cdot d}\right)}{2}\right)}.$$

The values of the key geometric parameters, measured on the longitudinal section of the compression cavity are given in Table 1, Table 2, Table 3 and Table 4 below.

### 2.3. Numerical Analysis

**Figure 4:**
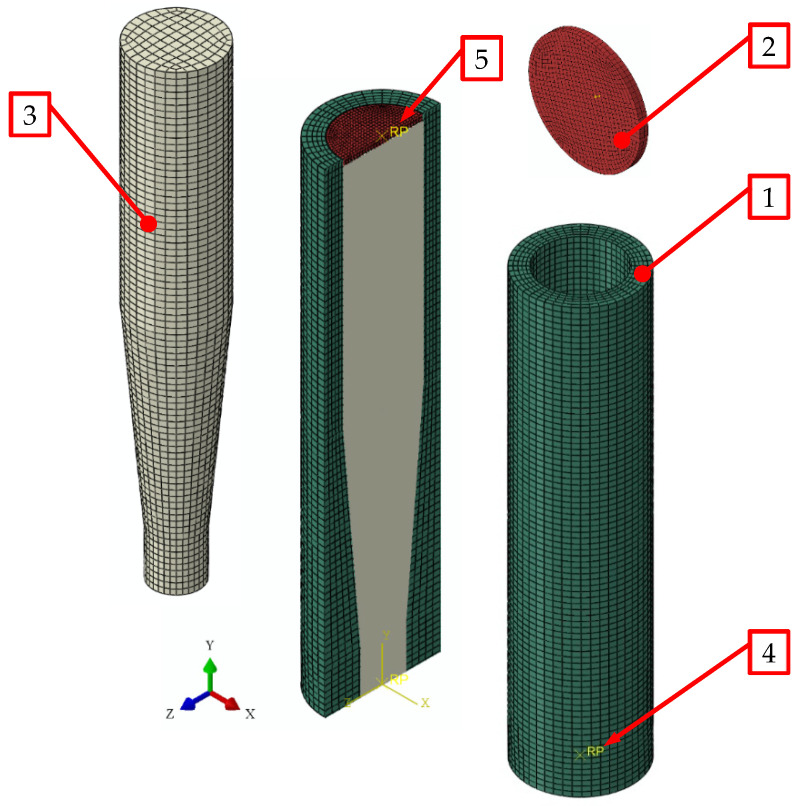
Discretization by finite elements of the model used in the numerical analysis of the process of extrusion: 1—single-hole die integrated with feed barrel, 2—ram, 3—solid, compressed carbon ice snow, 4—reference point on the die, 5—reference point on the ram.

The numerical model was prepared in Abaqus 2020 software by Dassault Systèmes, with the simulations performed in Abaqus/Explicit. Figure 4 shows the model of CS die with a FEM mesh superimposed on it.

The model is composed of three parts represented by finite elements (Figure 4). The main element is the single-hole die (1), integrated with the feed barrel to simplify the model, with a good effect on the uniformity of the finite element mesh. Inside the cylindrical part there is a compression ram (2), which moves by sliding with zero friction. The extruded material (3), i.e., dry ice snow, was modelled as an axisymmetric solid, corresponding in shape to the integrated single-hole die. Two reference points were defined:

− Reference point of the single-hole die (4), fixed on the die (1), and located in the bottom plane on the axis of symmetry;
− Reference point of the ram (5), fixed on the ram (2), and located on its top surface and on the axis of symmetry.

These reference points were used in the calculations to set the initial-boundary conditions relative to the ram and to the die.

The analysis was performed with the following parameters adopted for the respective aspects of the numerical model:

− Dynamic Explicit module analysis,
− Duration of analysis: t = 16 s.
− Mass Scaling feature was used to extend the step time to 0.0001 s. in order to improve the calculation efficiency.

**Figure 5:**
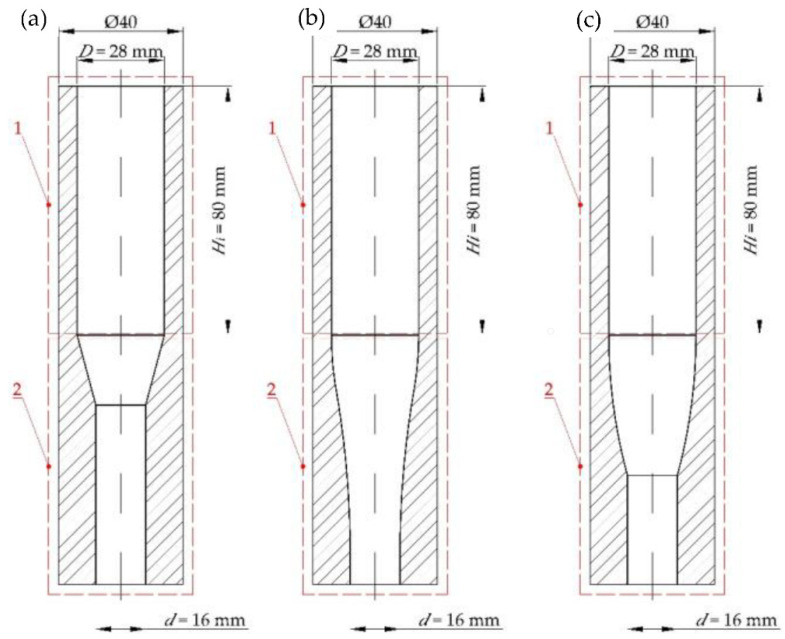
Shapes of integrated single-hole dies (examples): (**a**) CS incl. cylindrical section, (**b**) WKWP incl. cylindrical section, (**c**) WP incl. cylindrical section; 1—feed barrel area, 2—compression cavity area; *D*—feed barrel diameter, *H*—feed barrel height, *d*—outlet diameter commensurate to the diameter of the produced pellets.

The die model was designed to represent, as far as practicable, the actual extrusion conditions, and to this end:

− The model of the die integrated with the feed barrel was treated as a non-deformable body with a pre-defined and invariable geometry;
− The die was integrated with the cylindrical feed barrel of *D* = 28 mm in length and *H*_i_ = 80 mm in height (Figure 5);
− R3D4 4-node, 3-D, quadrilateral, and infinitely rigid elements used were distributed symmetrically about the central axis of the symmetry of the die. The finite elements were distributed evenly throughout the die and had an averaged edge length of 2 mm.

The same concept was applied in the model of the ram that pressed the material through the die. Its characteristic parameters were:

− Outside diameter of 27.5 mm and 2 mm thickness. This allowed the avoidance of friction between the ruled surface and the sides of the feed barrel. Furthermore, it was now possible to represent the process of extrusion with the ram moving inside a cylindrical chamber, on the specially built test bench.
− FEM mesh of R3D4 4-node, 3-D, quadrilateral, and infinitely rigid elements of 1 mm averaged edge length, uniformly sized over the whole extrusion ram surface.

**Figure 6:**
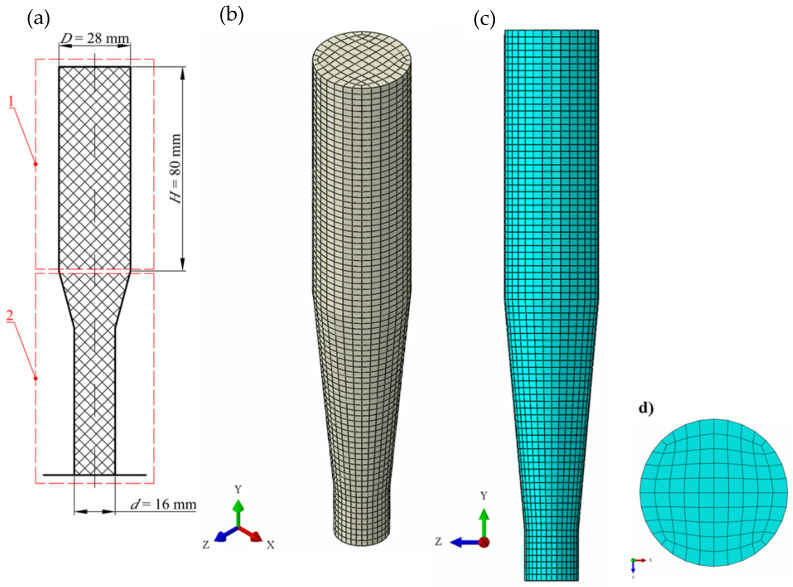
Shape and distribution of finite elements using an example of the compressed material model: (**a**) dimensions, (**b**) overview, (**c**) distribution of finite elements over a plane parallel to the axis of symmetry, (**d**) distribution of finite elements over a plane perpendicular to the axis of symmetry; 1—feed barrel area, 2—single-hole die area.

**Figure 7:**
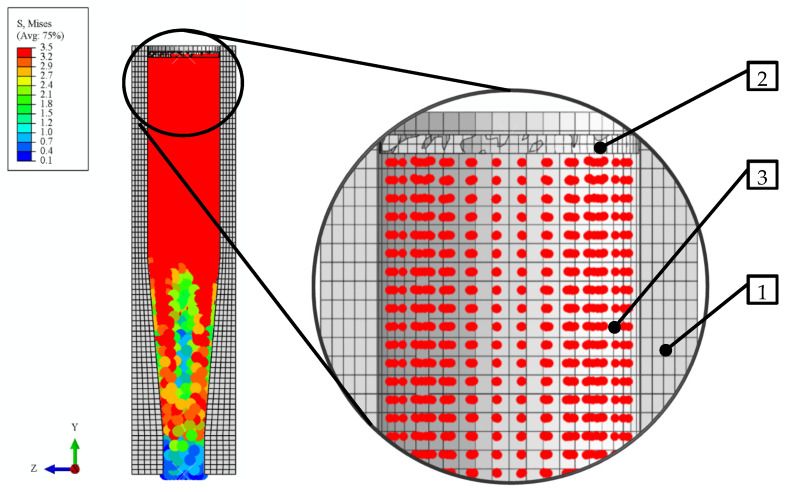
Distribution of particles in SPH analysis of the dry ice extrusion process: 1—integrated single-hole die, 2—ram, 3—particles of the extruded material.

A slightly different approach was applied in modelling the domain associated with the compressed dry ice (Figure 6):

− The compressed dry ice was modelled as a deformable elasto-plastic body;
− The shape and size of this domain was assumed to be commensurate to the internal cavity of the integrated die (Figure 5). This shape was assumed to change in the process of extrusion, as is typical of a deformable body;
− FEM mesh of C3D8R linear, 6-node, 3-D, reduced-integration elements. The FEM elements had 2 mm averaged edge length and were uniformly sized over the whole domain;
− SPH based approach was applied to define the properties of the compressed dry ice to allow the use of the SPH method. This ensures undisturbed flow of the compressed material through the die, leaving out nodal interactions. Thus, the individual finite elements are modelled as non-interacting particles (Figure 7).

**Figure 8:**
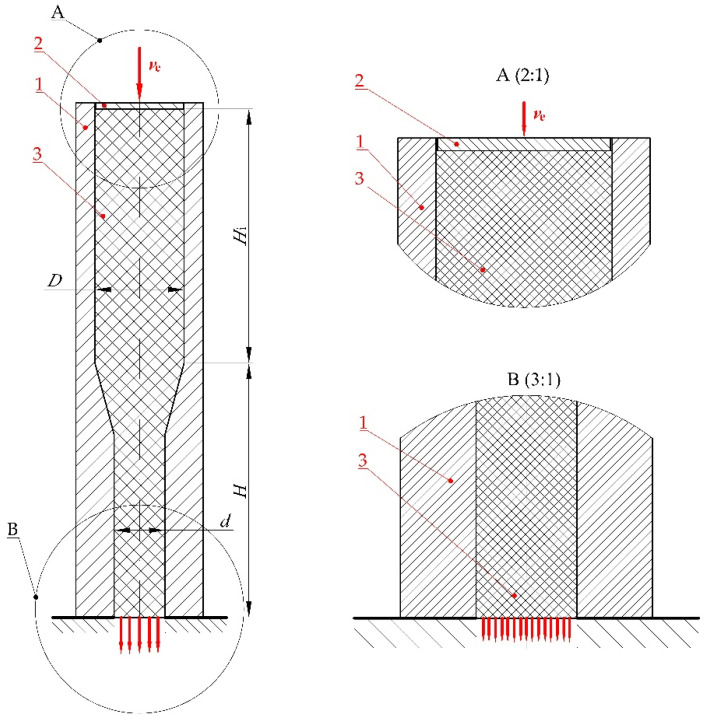
Schematic of the extrusion process during numerical analysis: 1—single-hole die integrated with feed barrel, 2—ram, 3—compressed dry ice snow, *v*_e_—extrusion speed, *D*—inlet diameter, *d*—outlet diameter, *H*—overall height of the die, *H*_i_—initial height of the extruded material.

The following input assumptions were set in the numerical simulation:

− The die was modelled as a non-deformable body with a pre-determined, fixed geometry;
− Full fixity boundary condition was assigned at the reference point (No. 4 in Figure 4) (constrained in all directions),
− At the ram reference point (No. 5 in Figure 4) a displacement of *s* = 80 mm over the whole period of analysis of *t* = 16 s. was assigned as the initial-boundary condition thus representing the empirical tests with extrusion speed of *v*_e_ = 5 mm/s (Figure 8);

The following contact conditions were assigned between all contacting surfaces:

− Tangentially: μ = 0.1 friction, in the direction normal to the surface—no penetration;
− With infinitely rigid walls of the integrated single-hole die (1) and ram (2) and “no penetration” boundary condition in place, the compressed dry ice (3) devoid of such conditions flew out through the opening in the bottom of the integrated die.

### 2.4. Experimental Verification

**Figure 9:**
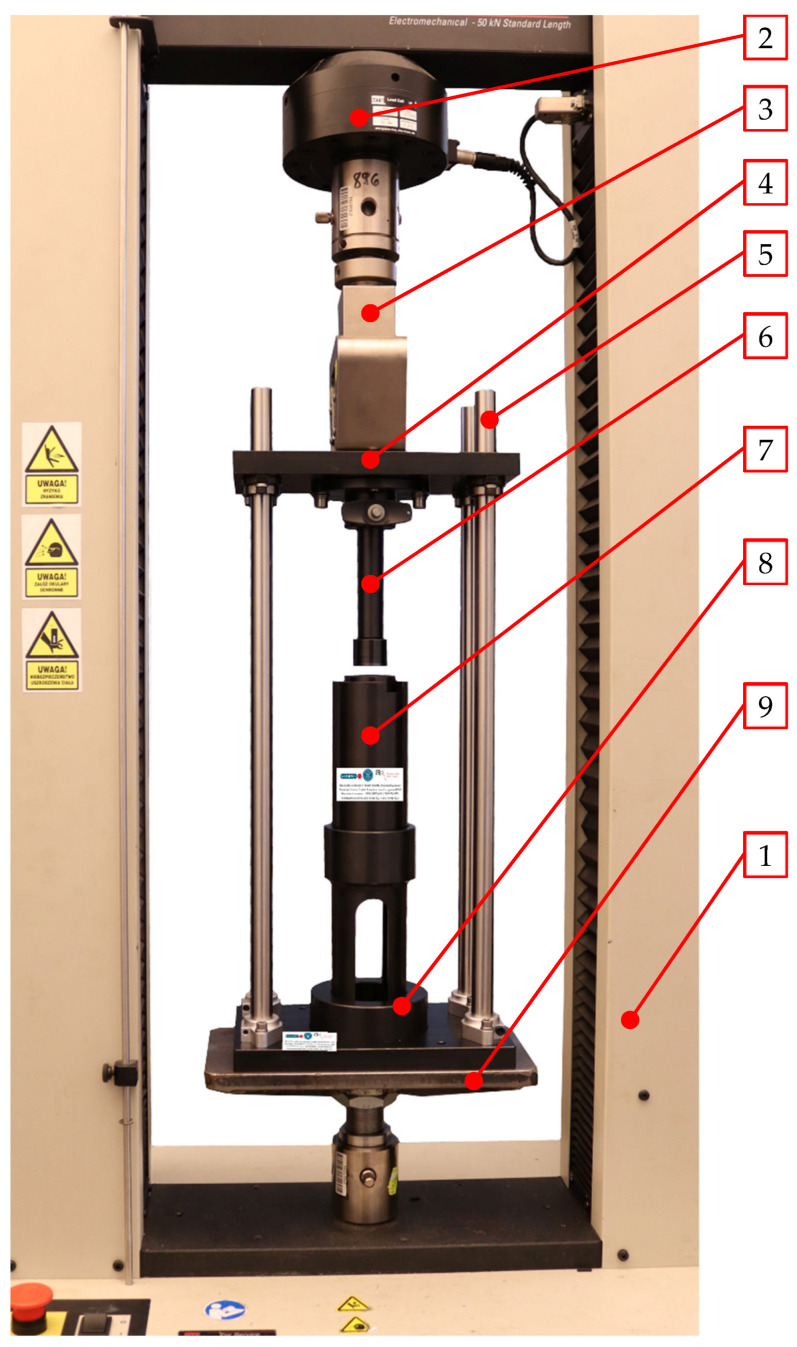
Test set-up used in this research for experimental verification of the compression and extrusion forces for the tested dies: 1—MTS Insight 50 kN test frame, 2—load cell, 3—grips of the moving crosshead, 4—ram mounting plate, 5—linear guides, 6—compression ram, 7—compression barrel assembly, 8—barrel base plate, 9—bottom support.

The experimental tests were done using a test frame mounted on a specially designed test bench (Figure 9). This test set-up included the MTS Insight 50 kN (MTS Systems Corporation, Eden Prairie, MN, USA) test frame (1), and the ram mounting plate, (4) supported by linear guides (5), was attached to the load cell (2) and grips (3) assembly. These guides are connected to the base (8), resting on the bottom support (9). The ram (6), which interacts with the compression barrel assembly (7), is fixed to the ram mounting plate (4). This assembly is composed of the feed barrel integrated with the compression cavity (1 in Figure 1), including the die (3 in Figure 1).

In the experimental tests, loose dry ice snow, of a known weight, was forced through the assembly by the movement of the MTS crosshead displaced with a uniform speed. The observed parameter was the force vs. displacement.

The test procedure included the following conditions:

− After every three test measurements, the compression barrel and ram were cooled in a container with dry ice at 195 K in order to reduce sublimation, during the process of extrusion, which could increase due to the ambient temperature of the laboratory (ca. 293 K), which was higher than the temperature of dry ice (195 K);
− Coaxial alignment of the ram, feed barrel, and compression cavity to ensure no contact between the ruled surface of the ram and the feed barrel opening. The ram diameter was 2% smaller than the feed barrel diameter to avoid the risk of the metallic surfaces coming in contact due to thermal expansion;
− The die was filled up with dry ice pellets before the first extrusion cycle, and after each cooling of the compression assembly, in order to stabilise the distribution of stress (the value measured in the first cycle was always left out);
− Constant speed of extrusion of *v*_e_ = 5 mm/s.;
− 31 ± 1 g of dry ice snow was fed into the feed barrel each time;
− Measurement of the applied force to an accuracy of 0.5% of the maximum measurement range of the test frame (0.5 accuracy class of the load cell fitted on the MTS test frame).

**Figure 10:**
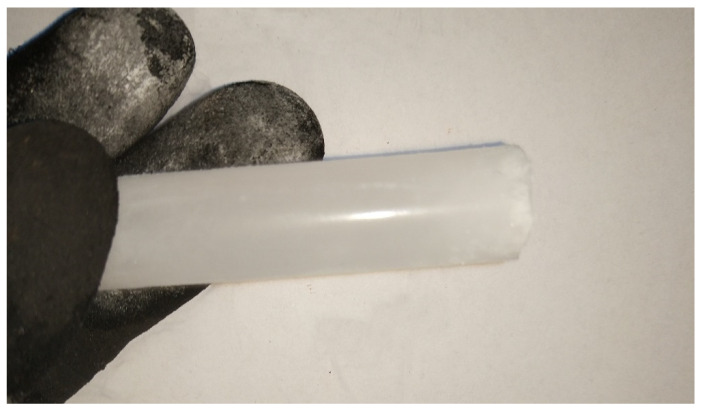
Sample of extruded material of uniform appearance and uniform density distribution.

The cohesion of the produced pellets was assessed through sensory analysis, with the criterion being the lack of cracking, and glassy structure over the entire pellet (Figure 10). The extrusion producing pellets affected by cracking or visible disfigurement were rejected. The test was repeated 16 times for each shape of the compression cavity, with each case producing acceptable pellets.

#### Statistical Analysis of the Test Data

One-way analysis of variance was performed using the post hoc Tukey’s test. Two populations of test data were compared within each type of die, i.e., CS, WK, WP, WKWP. The analysis was carried out using Statistica (version 13.3, TIBCO Software Inc., Palo Alto, CA, USA). All the comparisons were one-way ANOVAs, with statistical significance defined by the value of *p* < 0.05.

## 3. Discussion

**Figure 11:**
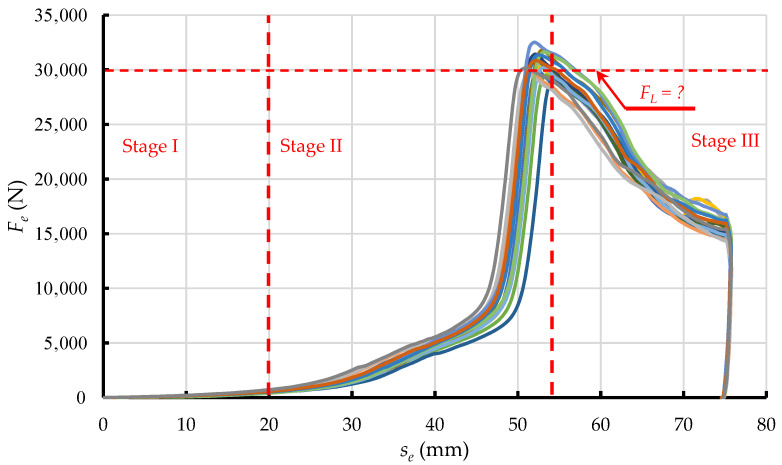
Relationships of the resistance force *F*_e_ vs. ram displacement *s_e_* during extrusion of dry ice snow through WKWP70-50 single-hole die, showing division into phases of the process (different colours means the following samples).

Examples of the resistance force *F*_e_ vs. displacement curves obtained with the above-mentioned test set-up are presented in Figure 11. Considering a relatively small scatter of data, these curves, based on their shape, can be sub-divided into phases, as was the case in previous studies (Figure 2), which attests to the accuracy of representation of the already used test method [21].

ANOVAs were carried out for the observed data for each die type, in turn. As the first step, normality of the respective populations was checked using the Shapiro-Wilk test, taking into account only the maximum extrusion resistance force *F*_L_. The minimum value of *p* was 0.069 or more.

Next, homogeneity of variances in comparable populations was checked for the respective die shapes. To this end, the value of *p* was determined using the Brown-Forsythe test. The hypothesis of the homogeneity of variance was confirmed as the obtained values exceeded the 0.05 criterion.

In the final step of ANOVA, the value of *p* was calculated for the cases which obtained a value of index below 0.05. This allowed the rejection of the hypothesis that compared dies had the same value of the parameter under analysis, i.e., the extrusion resistance force *F*_L_. This supports the hypothesis with statistically significant differences between the compared populations.

Tukey’s post hoc test was used to determine the statistical significance of the differences between the respective populations, the results of which are presented in Table 5, Table 6, Table 7 and Table 8. Based on the data presented in Table 8 we see that in the WKWP group statistical significance is observed only between the WKWP70-50 and WKWP70-25 populations. The other pairwise comparisons had a probability of error higher than 5%, and thus should not be made.

**Figure 12:**
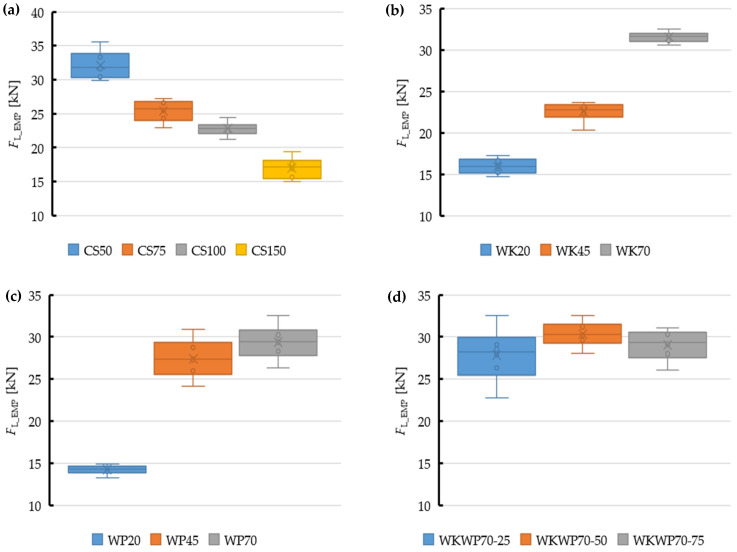
Box plots relating the median of the maximum observed extrusion force *F*_L_EMP_ with statistical data obtained for the dies under analysis, i.e., CS (**a**), WK (**b**), WP (**c**) and WKWP (**d**).

The key statistical data obtained for the respective groups of dies are compiled in Table 8 and illustrated on the box plots in Figure 12, relating the median of the maximum observed extrusion force *F*_L_EMP_ with the associated key statistical data.

The medians, which slightly differed from the relevant average values, were used in the analysis of differences between the observed and predicted data.

**Figure 13:**
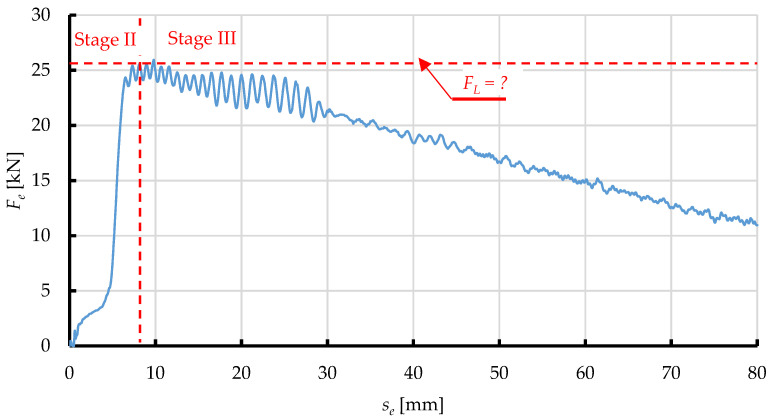
An example of the *F*_e_ vs. *s*_e_ relationship during extrusion of dry ice snow through a conical-cylindrical die (CS10), with marked division into phases.

Figure 13 shows an example of a numerical simulation of the process of extrusion of dry ice snow as a *F*_e_ vs. *s*_e_ relationship. As it can be seen on the graph, two phases of the simulated process can be distinguished, which can be assigned to the relevant experimental data:

− Phase 2, in which the value of *F*_e_ decreases due to elastic strain of the material and its frictional interaction with the internal surfaces of the compression cavity. This increase continues up to the maximum compression force *F*_L,_ which marks the commencement of extrusion,
− Phase 3, in which the value *F*_e_ decreases due to the plastic strain of the extruded material, whose value depends on the shape of the compression cavity.

Note that this simulation with elasto-plastic model lacks a distinguishable Phase 1, identified during actual extrusion of the material. This is due to the simplification characteristics of the adopted elasto-plastic model, where the material is modelled as compressed right from the start, thus leaving out the effect of compression. That said, the maximum force applied to extrude the material through the die *F*_L_ is the most interesting parameter at this stage of research.

An interesting phenomenon is the oscillation of the value of the *F*_e_ material force by the matrix, observed at the end of stage 2 and throughout Stage 3, while after exceeding the displacement *s*_e_ about 30 mm, this oscillation significantly reduces its amplitude. This phenomenon is due to the stick-slip effect, which is associated with the elasto-plastic representation of the extruded material. In the area of pure occurrence of this phenomenon (approx. 8 mm < *s*_e_ < approx. 30 mm), the extruded material does not extend from the matrix at total speed, equal to the speed of the piston movement *v*_e_. In this respect, *s*_e_ displacement, due to the elastic deformation of its volume, the speed of the material flowing from the matrix is significantly lower. Considering the coincidence of this phenomenon, with friction of the material on the walls of the matrix and the internal friction of the deposit, are good conditions for the resulting effect of the stick-slip effect-i.e., periodic fluctuations in movement speed, as a result of the mutual impact of friction force and force responsible for pressing. The reduction of its intensity (decrease in the amplitude of the oscillation of force) is present after exceeding a certain displacement (*s*_e_ = approx. 30 mm), when a portion of the compressed material (which is deformed in an elastic way), which is initially placed in front of the coincidence, begins to fill its volume. At the same time, the material originally present, leaves the matrix. Therefore, part of the material previously deformed in an elastic way (already in the range of plastic deformation) is less susceptible to this effect.

Therefore, this parameter was analysed using the data predicted by the numerical simulation *F*_L_FEM_ and the median of the experimental data *F*_L_EMP_. The results are compiled in Table 9 below.

**Figure 14:**
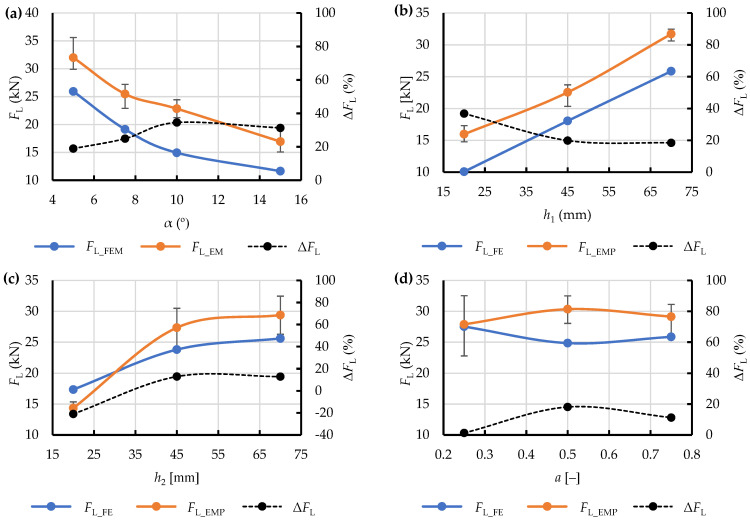
Comparison of the curves representing the relationship of the maximum force during dry ice extrusion and the adopted variable geometric parameters of the analysed single-hole dies for: maximum predicted force (*F*_L_FEM_), maximum observed force (*F*_L_EMP_) and their percent difference Δ*F*_L_ for the die groups CS (**a**), WK (**b**), WP (**c**) and WKWP (**d**).

The results are also represented graphically in Figure 14 as relationships of the maximum extrusion resistance force *F*_L_, both *F*_L_FEM_ (predicted) and *F*_L_EMP_ (observed), and the relevant geometric parameters of the respective die shapes (Table 1, Table 2 and Table 3). A relatively high similarity of the obtained curves was observed in terms of monotonicity. Unfortunately, the values noticeably differ between the test points in all cases. This can be explained by a fixed value of yield stress *σ*_pl_ used in all the numerical simulations, which was determined experimentally on compressed material. This implies inadequacy of the applied procedure as in the analysed system yield stress apparently depends on the type of die used in the process. However, this assumption appears correct as long as the above-mentioned value is taken as a process-specific parameter rather than an intrinsic property of the material.

This hypothesis needed to be verified. To this end, at all the test points (i.e., the values of the geometric parameters of the respective dies and their corresponding values of *F*_L_FEM_ and *F*_L_EMP_) mathematical functions were derived, describing the percent difference between the obtained values in relation to the observed extrusion resistance:

(8) $$\Delta F_{L}=\frac{F_{L\_EMP}-F_{L\_FEM}}{F_{L\_EMP}}\cdot 100\%,$$

and were related to the geometric parameters of the dies under analysis (Figure 14). The relationship obtained in this way showed a difference reaching as much as 40%, which excludes the data predicted with this numerical model from further analysis and, more importantly, from further work on a simplified model of the dry ice extrusion process with the application of SPH.

However, this difference served to derive the corrective function for the yield stress *σ*_pl_ used in the numerical elasto-plastic model from the following equation:

(9) $$\sigma_{pl\_COR}=\sigma_{pl}\cdot \frac{1+\Delta F_{L}}{100},$$

**Figure 15:**
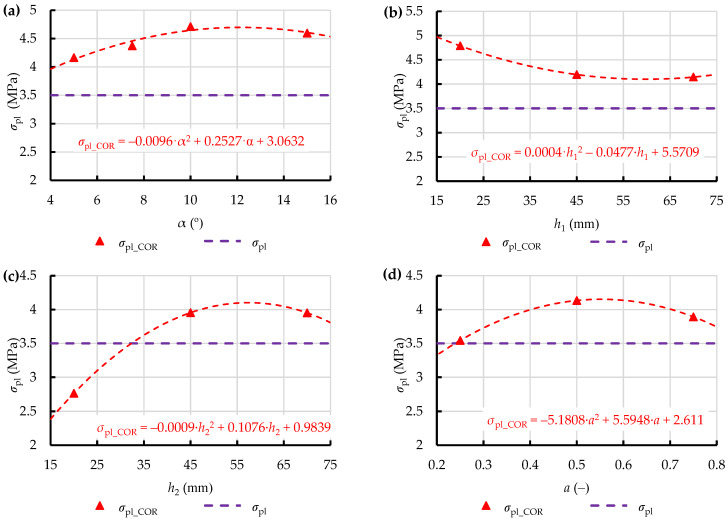
Values of the corrected yield stress of compressed dry ice snow *σ*_pl_COR_ depending on the geometric parameters of single-hole dies, and their comparison with the constant *σ*_pl_ for dies of groups CS (**a**), WK (**b**), WP (**c**), and WKWP (**d**).

In this way, corrected yield stresses *σ*_pl_COR_ were obtained for the test points set on all the analysed dies (Figure 15). Relationships describing the variation of this parameter as a function of the key geometric parameters of the analysed dies, were derived in addition, considering the future application of this approach in optimisation of the geometric parameters of extrusion dies over the entire variation domain.

**Figure 16:**
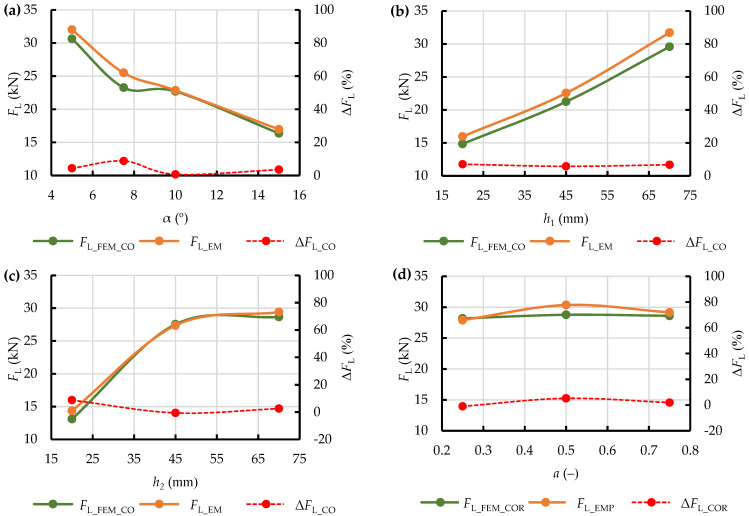
Comparison of the curves representing the relationship of the maximum force during dry ice extrusion and the adopted variable geometric parameters of the analysed single-hole dies for: corrected maximum predicted force (*F*_L_FEM_COR_), calculated with the corrected yield stress *σ*_pl_COR_, maximum observed force (*F*_L_EMP_), and their percent difference Δ*F*_L_COR_ for the die groups CS (**a**), WK (**b**), WP (**c**), and WKWP (**d**).

The obtained corrected yield stress values *σ*_pl_COR_ were used in the repeated numerical calculations performed with the use of the SPH method. Other settings remained unchanged. The results of the comparison between the corrected extrusion resistance force *F*_L_FEM_COR_ and the observed values of this force *F*_L_EMP_ are shown in Figure 16, together with the percent difference between these two Δ*F*_L_COR_. As it can be seen, this treatment improved the accuracy of representation of the process by numerical analysis, with regards to the obtaining of the maximum extrusion resistance force. After correction of the initial yield stress the percent difference did not exceed 10%, and the curves of *F*_L_FEM_COR_ and *F*_L_EMP_ became more similar in terms of monotonicity (as compared to the curves obtained for the constant value of *σ*_pl_).

## 4. Conclusions

The main purpose of the on-going research by the authors is to develop a relatively simple technique of estimating the maximum resistance during compression of solid dry ice that could be used in the design of extrusion dies, helping to improve the efficiency of the extrusion process, i.e., to minimise the input of energy while ensuring high quality product. The above-mentioned overall objective motivated the authors to look for an appropriate numerical modelling method that could be used in this connection. Therefore, it was assumed that it would be possible to represent dry ice with an elasto-plastic material assigned with the properties of dry ice pellets, thus avoiding complex models used in simulations of the process of compression. This approach also minimises the number of material properties that must be determined experimentally as an input for the calculations.

The FEM model prepared as part of this research represents the extrusion of dry ice snow through the die, with omission of the compression phase, designated here as Phase 1. Still, owing to the correct representation of phases 2 and 3, this approach can be successfully used in research dealing with the determination of the maximum compression resistance. Therefore, we can conclude that for this aspect (determination of the maximum extrusion resistance), the process of compression may be simulated by extrusion of compressed material through the die.

Yet another issue to be dealt with is the adoption of an appropriate parameter defining the plastic strain of the domain extruded through the die. The proposed SPH model with the adopted constant yield stress *σ*_pl_ (determined by testing the compressed material) failed to accurately predict the maximum extrusion resistance force for the different die parameters. To cope with this problem, yield stress was treated as a process specific variable, depending on the geometric parameters of the die rather than a constant property of the extruded material. The corrected yield stress *σ*_pl_COR_ variable in the domain of geometric parameters of the die was used, and the corrected FEM model reduced the difference between the predicted and observed values to less than 10%, thus solving the problem. Being a gross simplification of the actual process of extrusion, with the above-mentioned level of error, the accuracy of the proposed elasto-plastic model with the use of SPH should be considered satisfactory. This is especially true when the focus is on the maximum extrusion force rather than on the simulation of the entire process, as it is the case in this research. In addition, the tests performed on dry ice snow do not yield stable results, mainly due to technical constraints, with sublimation of this material being the main challenge. This is evidenced by the problem with reducing the probability of error to an acceptable level when comparing the subsequent data populations. This problem observed in laboratory experiments also applies to dry ice production on an industrial scale, i.e., under generally less controlled conditions.

Considering the above constraints, it is reasonable to conclude that dry ice may be correctly represented by elasto-plastic material in numerical simulations of the process of dry ice extrusion for the purposes of studies on the efficiency of this process. Furthermore, subject to appropriate input assumptions (value of the yield stress parameter), the above simulation can yield satisfactory results confirmed by experimental data. The authors plan to apply this approach to modelling the process of extrusion in future research, analysing the influence of the shape of the die cavity on the energy requirements of the process (by analysing the resistance force), over continuously varying domains, covering the geometric parameters of the die and the associated shape optimisation efforts. However, an additional parameter should be introduced for this purpose, that would allow verification of the quality of the produced pellets.

## Figures and Tables

**Table 1:** Geometric parameters of CS dies.

Designation	Taper Angle *α* (°)	Length of Conical Section *h* (mm)
CS50	5	68.58
CS75	7.5	45.57
CS100	10	34.03
CS150	15	22.39

**Table 2:** Geometric parameters of WK and WP dies.

Designation	Rounding Radius *R*_1_ (mm)	Length of Spherical Section *h*_1_ (mm)
WK20/WP20	36.3	20
WK45/WP45	171.8	45
WK70/WP70	411.3	70

**Table 3:** Geometric parameters of WKWP dies.

Length of the Convex/Concave Section *H’*, mm	Designation	Proportionality Coefficient *a* (−)	Rounding Radius *R*_1_, mm	Length of Spherical Section, *h*_1_, mm	Rounding Radius R_2_, mm	Length of Spherical Section, *h*_2_, mm
70	WKWP70-25	0.250	102.8	17.5	308.5	52.5
	WKWP70-50	0.500	205.7	35.0	205.7	35.0
	WKWP70-75	0.750	308.5	52.5	102.8	17.5

**Table 4:** Results of the Tukey’s post hoc test for the CS group of dies.

	CS50	CS75	CS100	CS150
CS50	-	0.000157	0.000164	0.000157
CS75	0.000157	-	0.000157	0.000157
CS100	0.000164	0.000157	-	0.000157
CS150	0.000157	0.000157	0.000157	-

**Table 5:** Results of Tukey’s post hoc test for the WK group of dies.

	WK20	WK45	WK70
WK20	–	0.000124	0.000124
WK45	0.000124	–	0.000124
WK70	0.000124	0.000124	–

**Table 6:** Results of Tukey’s post hoc test for the WP group of dies.

	WP20	WP45	WP70
WP20	–	0.000118	0.000118
WP45	0.000118	–	0.000333
WP70	0.000118	0.00333	–

**Table 7:** Results of Tukey’s post hoc test for the WKWP group of dies.

	WKWP70-25	WKWP70-50	WKWP70-75
WKWP70-25	–	0.000324	0.082838
WKWP70-50	0.000324	–	0.091569
WKWP70-75	0.082838	0.091569	–

**Table 8:** The key statistical data.

	*F*_L_^Min^ (N)	*F*_L_^Q1^ (N)	*F*_L_^Q2^ (N)	*F*_L_^Q3^ (N)	*F*_L_^Max^ (N)	*F*_L_^AVG^ (N)
CS50	29,910	30,400	31,582	33,297	35,602	32,003
CS75	22,898	24,345	25,990	26,590	27,190	25,486
CS100	21,237	22,425	22,890	23,050	24,442	22,840
CS150	15,065	15,648	17,355	17,652	19,362	16,948
WK20	14,768	15,279	15,973	16,642	17,318	15,973
WK45	20,326	22,400	23,100	23,322	23,714	22,549
WK70	30,602	31,618	31,851	31,156	32,472	31,708
WP20	13,342	14,065	14,377	14,599	14,951	14,348
WP45	24,217	25,962	27,332	28,776	30,917	27,350
WP70	26,339	28,295	29,482	30,264	32,520	29,396
WKWP70-25	22,763	26,326	28,522	29,079	32,527	27,890
WKWP70-50	28,038	29,627	30,216	31,207	32,502	30,352
WKWP70-75	26,070	28,014	29,580	30,323	31,114	29,142

**Table 9:** Maximum predicted extrusion force *F*_L_FEM_ and its average value calculated from the experimental data *F*_L_EMP_.

Die Designation	Predicted Maximum Extrusion Force *F*_L_FEM_ (N)	Average Maximum Extrusion Force Calculated from the Experimental Data *F*_L_EMP_
CS50	25,934	31,582
CS75	19,137	25,990
CS100	14,943	22,890
CS150	11,637	17,355
WK20	10,091	15,973
WK45	18,062	23,100
WK70	25,861	31,851
WP20	17,358	14,377
WP45	23,802	27,332
WP70	25,604	29,482
WKWP70-25	27,533	28,522
WKWP70-50	24,853	30,216
WKWP70-75	25,871	29,580

## Data Availability

Not applicable.
